# *In Vitro* and *In Vivo* Studies on the Effects of Bone Morphogenetic Protein-7 on Human Kidney and Lung Tumor Cells

**Published:** 2010-09

**Authors:** Lee-Chuan C. Yeh

**Affiliations:** *Department of Biochemistry, The University of Texas Health Science Center at San Antonio, San Antonio, TX, USA*

**Keywords:** BMP-7, BMP receptors, cell proliferation, human kidney and lung tumor cell lines, tumor growth *in vivo*

## Abstract

Breast, kidney, lung, and prostate cancers are among the human cancers that show high propensity to form bone metastasis. Bone morphogenetic protein (BMP) -2 and -7 are two members of the BMP superfamily which show the most potent biological activity in stimulating bone differentiation and repair. These proteins have been used in clinical treatment of orthopedic diseases and have also been studied in different types of cancer. We report here detection of mRNA coding for three type I and one type II BMP receptors in G-402 kidney tumor cells and A-549 lung tumor cells, suggesting that these cells are responsive to BMPs. We then observed that BMP-7 inhibited cell proliferation of both cell lines in a protein concentration dependent manner *in vitro*. Additionally, when BMP-7-treated cells were implanted into the flank region of male nude mice, smaller tumors, compared to those formed with the untreated cells, were observed. Histological analysis showed that the masses formed at the site of implantation displayed significantly less number of tumors than the control and exhibited significant ectopic bone formation. These findings raise the possibility of BMP-7 as a therapeutic agent for kidney and lung cancers.

## INTRODUCTION

Bone morphogenetic proteins (BMPs) are members of the transforming growth factor-β (TGF-β) superfamily. They were first identified based on their ability to stimulate bone formation but were subsequently shown to play a major role in development, differentiation, and apoptosis of other tissues (1-6). Based on their amino acid sequence homology, BMPs are divided into several subfamilies, such as the BMP-2 and -4 subfamily, the BMP-5, -6 (Vgr1), -7 (OP-1), and -8 (OP-2) subfamily, and the GDF-5/CDMP-1, -6/CDMP-2, and -7 subfamily. BMPs exert their biological effects by binding to two structurally similar, single-pass transmembrane receptors, classified as type I and II (7-12). Four mammalian type I (ActR-IA/ALK-2, ActR-IB/ALK-4, BMPR-IA/ALK-3, BMPR-IB/ALK-6) and three type II (ActR-IIA, -IIB, and BMPR-II) receptors have been shown to bind BMPs. Binding of ligand to the receptor results in receptor phosphorylation and activation of a subset of Smad proteins leading to the formation of an active protein complex. The complex translocates to the nucleus and regulates target gene transcription. BMPs can also activate other signaling pathways, e.g. MAP kinase, JAK-STAT and Wnt pathways (13).

Due to their diverse biological activities, BMPs are relevant to cancer, particularly in those cancers that show high propensity to form bone metastasis. Among them are breast, kidney, lung, and prostate cancers. Of the BMPs, BMP-2 and -7 are the two members which show the most potent biological activity in stimulating bone differentiation and repair. These proteins have been used in clinical treatment of orthopedic diseases and have also been the subject of study in cancer. Whereas numerous studies have been conducted on the effects of BMP-7 on breast and prostate cancers, little has been reported on the effects of BMPs in general and BMP-7 in particular on lung and kidney cancer to date. Buckley *et al* (14) reported that BMP-4 stimulated A-549 lung cell senescence *in vitro* and the treated cell was less tumorigenic when implanted in nude mice. Tada *et al*. (15) reported that BMP-2 suppressed A-549 cells. On the other hand, Langenfeld *et al*. (16) reported that BMP-2 stimulated growth, invasiveness and migration of A-549 cells. BMP-6 did not appear to have any effect on three human renal carcinoma cell lines 112, 117, and 181, seemingly due to a decreased BMPR-II expression (17). Basic-Jukic *et al*. (18) reported increased BMP-6 mRNA in patients with human clear cell renal carcinoma (CCRC) compared to normal individuals. No significant correlation was found between BMP-6 expression level and disease presentation and progression. On the other hand, Higinbotham *et al*. (19) reported that the BMP-7 expression level was lower in nephroblastomas.

In the present study, we first examined expression of BMP receptors in kidney (G-402) and lung (A-549) tumor cell lines. Having demonstrated expression of BMP receptors in these cell lines, we then examined the effects of BMP-7 on their proliferation *in vitro* and tested the tumorigenicity of BMP-7-treated cells implanted in nude mice.

## MATERIALS AND METHODS

### Materials

Recombinant human BMP-7 was provided by Stryker Biotech (Hopkinton, MA) and was dissolved in 47.5% ethanol/0.01% trifluoroacetic acid. FBS, PBS and media were purchased from Life Technologies (Grand Island, NY). TRI Reagent was purchased from Sigma (St. Louis, MO). Radioisotopes were purchased from ICN (Irvine, CA). All reagents were of molecular biology grade and all buffers were prepared with diethylpyrocarbonate-treated water.

### Cell culture

Both tumor cell lines were purchased from the American Tissue Culture Collection (ATCC): kidney G-402 (CRL-1440, Lot #203818) and lung A-549 (CCL-185, Lot #2169440). G-402 was cultured in McCoy’s 5α+10% FBS and A-549 was cultured in Ham’s F12K+10%FBS in the presence of penicillin/streptomycin at 37°C in a humidified 5% CO_2_ atmosphere. Media were replenished every 3 days.

### Northern blot analysis

Total RNA was isolated from cells cultured in D-100 tissue culture dishes using the TRI reagent (Sigma, St. Louis, MO) following the manufacturer’s recommendation. The intactness of the RNA preparation was examined by agarose (1%) gel electrophoresis followed by ethidium bromide staining. Only RNA preparations showing intact species were used for subsequent analyses. Northern blot analysis was used to probe for the presence of mRNA for ActR-I, BMPR-IA, BMPR-IB, and BMPR-II as previously described (20). The cDNA probes for ActR-I, BMPR-IA, BMPR-IB, and BMPR-II were obtained by digestion of the corresponding plasmids with the appropriate restriction endonucleases as reported previously (20). Specifically, the 580-bp ActR-I insert was obtained by digestion of the parent plasmid containing the ActR-I insert with EcoRI/AvaI. The 530-bp BMPR-IA insert was obtained by digestion with HindIII/PvuII. The 660-bp BMPR-IB insert was obtained by digestion with HpaI/SacI. The 800-bp BMPR-II insert was obtained by PstI digestion of hBMPR-II cloned in pCMV5. The resultant cDNA fragments were purified by agarose gel electrophoresis and were labeled with [α-^32^P]dATP using the Strip-EZ DNA labeling system (Ambion Co, Austin, TX). The labeled cDNA probes were purified through a Midi-SELECT G-25 spin column (IBI, New Haven, CT) to remove the un-incorporated nucleotides. The 18S rRNA was probed with a _32_P-labeled, 18S-specific oligonucleotide with the following sequence: 5’-GCCGTGCGTACTTAGACATGCATG-3’. Experiments were conducted 4 times.

### Thymidine incorporation

Cells were subcultured at a cell density of 2 × 10^4^/ml in a 48-well plate and grown in the appropriate medium with serum until mid-log. The specific day at which the culture reached mid-log (the doubling time) varied according to the individual cell line. Cells were then treated with various concentrations of BMP-7 (0, 0.1, 0.5, 1.0, 5.0, 10, 50, and 100 μg/ml) in serum free-medium containing 0.1% BSA for 18 h. Cell proliferation was measured by [^3^H]thymidine incorporation into DNA molecules. The extent of thymidine incorporation into DNA was determined as previously described (21). Briefly, cells were pulsed with [^3^H]thymidine (5 μCi/ml) for 6 h following BMP-7 treatment. After removal of the medium containing the unincorporated thymidine, cells were rinsed with cold 1X PBS. The radiolabeled DNA was precipitated by cold 10% TCA for 15 min, solubilized in 0.1N NaOH at 37°C for 10 min, and neutralized with 0.1N HCl. The amount of radioactivity was determined by scintillation spectrometry in the presence of Econo-Safe cocktail (5 ml). The rate of cellular proliferation of the BMP-7-treated samples was defined as a percentage of the solvent-treated control.

### *In vivo* tumor formation assay and histology

Two groups of homozygous male nude mice were used; each group consisted of eight mice. Mice in group 1 was injected with tumor cells alone (10^6^ cells grown to mid log phase in 100 μl PBS); and mice in group 2 was injected with tumor cells treated with BMP-7 (10^6^ cells grown to mid log phase in 100 μl PBS and treated with 100 μg/ml BMP-7 for 24 h). Each mouse received a single subcutaneous injection in the flank region. Mice were examined daily for general health and the development/progression of masses for 49 days after which they were necropsiesed. Masses at the injection sites of mice treated as described above were collected, fixed in 10% neutral buffered formalin, embedded in paraffin, section at 5μ, stained with hematoxylin and eosin (H&E) and examined microscopically.

### Statistical analysis

Data are presented as the mean ± SEM. Statistical differences between means were determined by one-way ANOVA.

## RESULTS

### BMP-7 on kidney tumor cell line G-402

**Figure 1:**
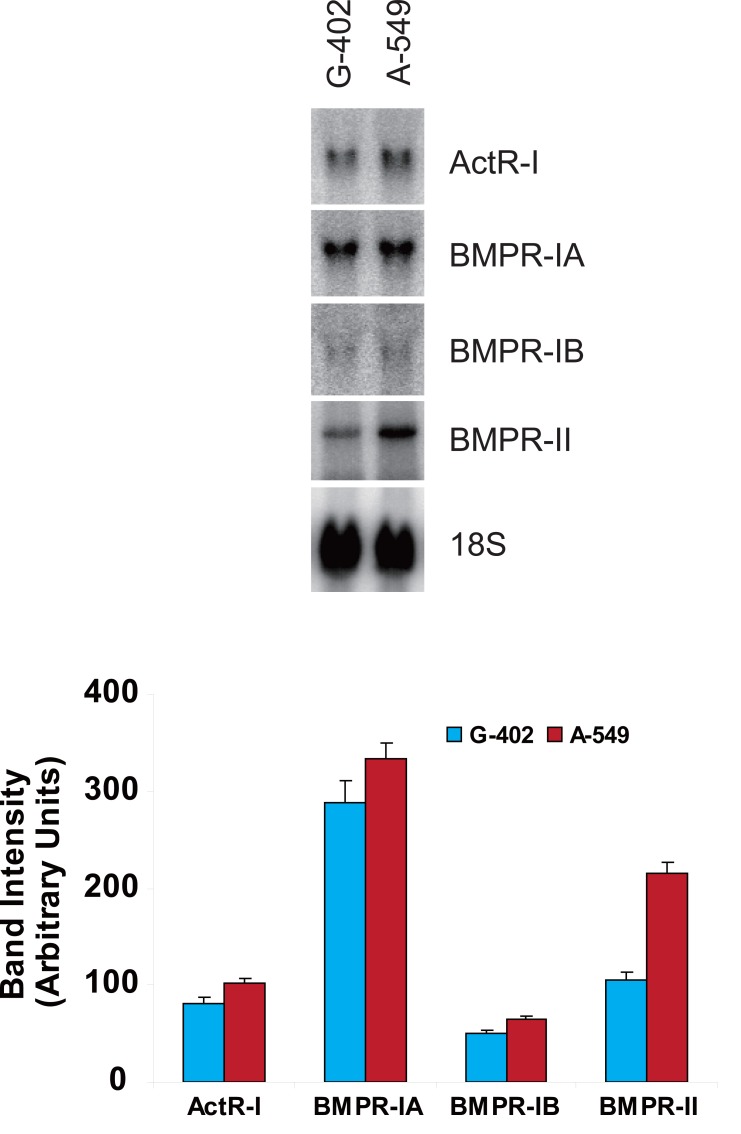
Messenger RNA expression of BMP receptors in kidney G-402 and lung A-549 cells in culture. Total RNA was isolated from cells and the levels of mRNA coding for the different BMP receptors were determined by Northern blot analysis. (A) Representative autoradiogram of Northern blots; (B) Quantitative values of ActR-I, BMPR-IA, -IB, and –II. Values represent the mean ± SD of 4 independent measurements.

Messenger RNAs coding for ActR-I, BMPR-IA, -IB, and -II were detected in the human kidney tumor cell line G-402 (Fig. 1A). Of the three type I receptors that are known to bind BMP, BMPR-IA mRNA was the most abundant followed by ActR-IA and BMPR-IB (Fig. 1B). The only type II BMP-binding receptor mRNA detected was that encoding BMPR-II.

**Figure 2:**
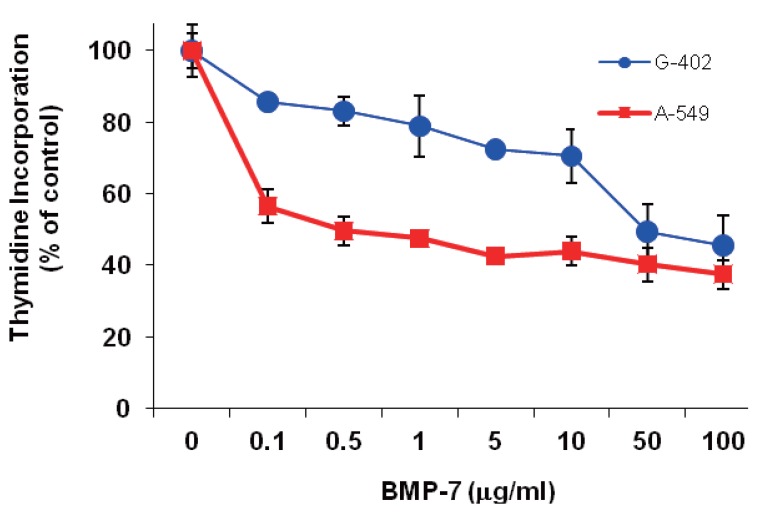
Effects of BMP-7 on thymidine incorporation in G-402 (●) and A-549 (■) in culture. Cell cultures were treated with different concentrations of BMP-7 for 18 h and 6 additional hour in the presence of [^3^H]thymidine. Level of radioactivity incorporated into TCA insoluble materials was measured and normalized to the vehicle-treated control experiment. Values represent mean±SD of 4 independent determinations.

BMP-7 inhibited thymidine incorporation in G-402 cells in a protein concentration dependent manner (Fig. 2). However, even at the highest protein concentration tested (10 μg/ml), only 55% (compared to the control) inhibition was achieved.

**Figure 3:**
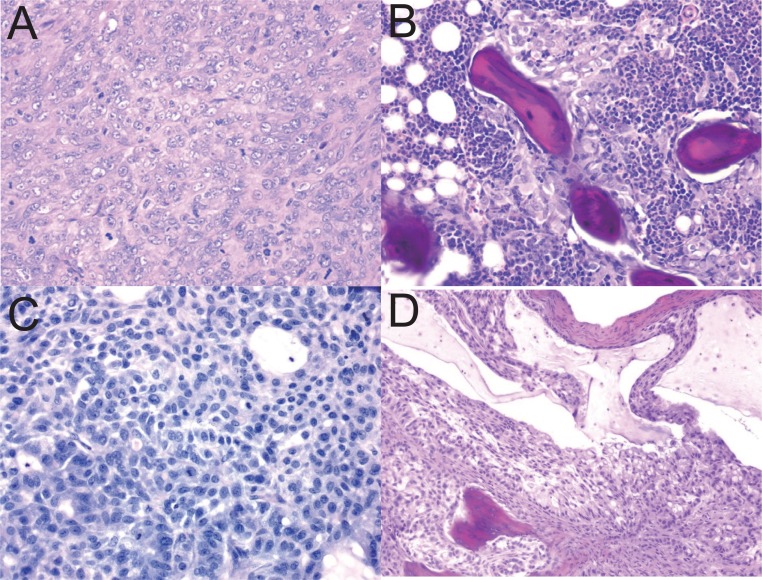
Sections from subcutaneous mass in the flank of nude mouse implanted with tumor cells G-402 or A-549 were stained with Hematoxylin and Eosin. Representative photographs of the sections are shown (200X magnification). (A) implanted with G-402, section contained neoplastic renal leiomyosarcoma cells; (B) implanted with G-402 treated BMP-7, mass comprised mainly of bone, bone marrow and foci of tumor cells; (C) implanted with A-549 cells, section contained neoplastic bronchiolar epithelial cells; (D) implanted with A-549 cells treated BMP-7, section showed neoplastic bronchiolar epithelial cells containing a focus of bone.

The effect of BMP-7 on tumor development was examined in a homozygous BALB/C nude mouse tumor xenograft model. Two groups of homozygous male mice were used: Group 1 received tumor cells alone in PBS, and group 2 received the same number of tumor cells but were treated with BMP-7 prior to implantation. Mice in both groups developed palpable masses. Mice receiving tumor cells alone (group 1) showed masses which had the typical and expected histological appearance of a sarcoma (renal leiomyoblastoma) (Fig. 3A). In contrast, all male mice implanted with BMP-7-treated G-402 cells developed masses that histologically were comprised of a nodule or shell of ectopic bone containing active bone marrow. Foci of tumor cells were detected within or adjacent to the ectopic bone focus in 6 of 8 mice from this treatment group. Tumor cell foci were small and well contained within the bone nodule. Fig. 3B shows the typical appearance of a nodule of mature bone containing tumor cells.

### BMP-7 on lung tumor cell line A-549

Messenger RNAs coding for ActR-I, BMPR-IA, -IB, and -II were detected in the human lung tumor cell line A-549 (Fig. 1A). Of the three type I receptors that are known to bind BMP, BMPR-IA mRNA was the most abundant following by ActR-IA and BMPR-IB. BMPR-II mRNA was the only type II BMP-binding receptor mRNA detected (Fig. 1B).

BMP-7 inhibited thymidine incorporation in A-549 cells in a protein concentration dependent manner (Fig. 2). The extent of inhibition was dramatic at low concentrations of BMP-7. Growth was reduced to about 50% of the control at 0.5 μg/ml. Higher BMP-7 concentrations did not further inhibit A-549 growth.

The effect of BMP-7 on tumor development was examined in a homozygous BALB/C nude mouse tumor xenograft model. Mice receiving tumor cells alone developed palpable masses which had the typical and expected histological appearance of a sarcoma (Fig. 3C). All male mice receiving BMP-7-treated A-549 cells also developed masses that histologically consisted of solid neoplasms consistent with the histological origin of the tumor cell line, lung carcinoma and were comprised of a nodule or shell of ectopic bone containing active bone marrow. Tumors were generally solid but had cystic foci containing mucinous material and areas of necrosis. Foci of tumor cells were detected within or adjacent to the ectopic bone focus in 6 of 8 mice from this treatment group. Tumor cell foci were small and well contained within the bone nodule (Fig. 3D).

## DISCUSSION

### BMPR expression in kidney and lung tumor cell lines

One of the regulatory steps of BMP functions involves their receptors (BMPRs). Mutations or derangements of BMPRs have been found in several human diseases, such as skeletal defects, familial primary pulmonary hypertension and cancer. Prior to examining the effects of BMP-7 on the kidney and lung tumor cell lines, we first established that mRNAs coding for all three type I and one type II receptor were expressed in both tumor cell lines. Except for BMPR-II, all three type I receptors were expressed to similar levels in the two cell lines. mRNA coding for ActR-IA was also detected in these cell lines (Table 1) and to the best of our knowledge, our study is the first to report such a finding. Relevant to our observation is that of Langenfeld *et al*. (16) who reported that the BMPR-IB and –II expression level was lower in the lung tumor cells than that of normal lung tissue, whereas the BMP-IA expression level was similar in both primary lung tumors and normal lung tissue.

**Table 1:** BMPR mRNA in kidney and lung tumor cell lines

Cell line	Receptor mRNA				
	ActR-IA	BMPR-IA	BMPR-IB	BMPR-II	Reference

Kidney					
G-402	+	+	+	+	Present study
RCC112	-	+	+	+	17
RCC117, 181	-	+	+	-	17
Lung					
A-549	+	+	+	+	Present study
	-	+	+	+	16
H7249	-	+	+	+	16

### *In vitro* and *in vivo* effects of BMP-7 on G-402 kidney tumor cells

The role of BMP-7 in renal development was first demonstrated in BMP-7 knockout mice that showed a steady cessation of nephrogenesis (22), but the role of BMP-7 in renal tumorigenesis however has not been well studied. Nevertheless, Kwak *et al*. (23), who examined the expression of BMP-4, -6, and -7 in 185 cases of human renal carcinomas, found that patients expressing BMP-7 appeared to survive disease-free better than those not expressing BMP-7. The same report further demonstrated that BMP-6, a close family member of BMP-7 inhibited human renal carcinoma cell line 112 in a dose dependent manner. In accordance with these studies are the present *in vitro* and *in vivo* results which consistently showed that BMP-7 inhibited G-402 cell proliferation in a protein concentration dependent manner. However, even at very high concentrations of BMP-7, inhibition was not complete. The reason(s) for this resistance or insensitivity to BMP-7 inhibition at high concentrations is not clear at present. It is possible that G-402 may be heterogeneous in nature such that a subpopulation is insensitive to BMP-7. The implanted BMP-7 treated G-402 cells not only showed decreased tumor formation at the site of implantation but also developed nodule of ectopic bone containing active bone marrow. In agreement with our finding is the case report describing detection of BMP-2 and ossification in renal cell carcinoma (24).

### *In vitro* and *in vivo* effects of BMP-7 on A-549 lung tumor cells

That BMPs are involved in lung development has been established in animal studies (25, 26), but their role(s) in tumoriogenesis is less clear. Although the present findings are consistent with several published studies, there are differences as well. It is conceivable that different BMPs, even closely related members may exert different effects. For example, the present *in vitro* and *in vivo* results consistently showed that BMP-7 inhibited A-549 cell proliferation in a protein concentration dependent manner. In contrast, Langenfeld *et al*. reported that BMP-2 stimulated A-549 cell invasiveness and migration *in vitro* and *in vivo* (16). The difference between the two findings may be attributed to the difference in BMPs used in the two studies even though the same cell type was used in both studies. Our study used BMP-7 and Langenfeld *et al*. used BMP-2 which shares 60% homology with BMP-7. On the other hand, BMP-4, a member of the same subfamily of BMP-2, induced cell senescence in long-term culture of A-549 and inhibited A-549 cell proliferation (14). Furthermore, in agreement with the current observations are several publications reporting that BMP-7 inhibited growth of thyroid and myeloma tumor cells (27, 28). In addition, implantation of the BMP-7-treated A-549 cells into nude mice resulted in ectopic bone formation surrounded by tumor cells. Consistent with our *in vivo* observation is the report by Usami *et al*. (29) who observed heterotopic bone formation and presence of immunoreactive BMP-2 protein within lung tumor cells in a 46-year ago male patient. Germane to the present finding is the report that BMP-7 did not inhibit TGFβ1-induced epithelial-mesenchymal transition in lung epithelial cells (30). The authors further suggested that the potential therapeutic impact of BMP-7 may be tissue restricted.

In summary, the present study showed that BMP-7 inhibited, though not completely, cell proliferation of G-402 and A-549 tumor cells and suppressed tumor formation with ectopic bone formation in a xenograft mouse model. These findings provide a justification for further studies on the prospect of BMP-7 as a therapeutic agent for treatment of kidney and lung cancers as well as the mechanism of inhibition of BMP-7 on these types of tumors.
